# Epidemiology of serogroup B invasive meningococcal disease in Ontario, Canada, 2000 to 2010

**DOI:** 10.1186/1471-2334-12-202

**Published:** 2012-08-29

**Authors:** Vica Dang, Frances B Jamieson, Sarah Wilson, Prasad Rawte, Natasha S Crowcroft, Karen Johnson, Raymond S W Tsang, Shelley L Deeks

**Affiliations:** 1 Public Health Ontario, Toronto, ON, Canada; 2 Dalla Lana School of Public Health, University of Toronto, Toronto, ON, Canada; 3 Department of Laboratory Medicine and Pathobiology, University of Toronto, Toronto, ON, Canada; 4 National Microbiology Laboratory, Public Health Agency of Canada, Winnipeg, MB, Canada

**Keywords:** Invasive meningococcal disease, *Neisseria meningitidis*, Serogroup B, Epidemiology, Surveillance, Ontario, Canada

## Abstract

**Background:**

Invasive meningococcal disease (IMD) caused by serogroup B is the last major serogroup in Canada to become vaccine-preventable. The anticipated availability of vaccines targeting this serogroup prompted an assessment of the epidemiology of serogroup B disease in Ontario, Canada.

**Methods:**

We retrieved information on confirmed IMD cases reported to Ontario’s reportable disease database between January 1, 2000 and December 31, 2010 and probabilistically-linked these cases to Public Health Ontario Laboratory records. Rates were calculated with denominator data obtained from Statistics Canada. We calculated a crude number needed to vaccinate using the inverse of the infant (<1 year) age-specific incidence multiplied by expected vaccine efficacies between 70% and 80%, and assuming only direct protection (no herd effects).

**Results:**

A total of 259 serogroup B IMD cases were identified in Ontario over the 11-year period. Serogroup B was the most common cause of IMD. Incidence ranged from 0.11 to 0.27/100,000/year, and fluctuated over time. Cases ranged in age from 13 days to 101 years; 21.4% occurred in infants, of which 72.7% were <6 months. Infants had the highest incidence (3.70/100,000). Case-fatality ratio was 10.7% overall. If we assume that all infant cases would be preventable by vaccination, we would need to vaccinate between 33,784 and 38,610 infants to prevent one case of disease.

**Conclusions:**

Although rare, the proportion of IMD caused by serogroup B has increased and currently causes most IMD in Ontario, with infants having the highest risk of disease. Although serogroup B meningococcal vaccines are highly anticipated, our findings suggest that decisions regarding publicly funding serogroup B meningococcal vaccines will be difficult and may not be based on disease burden alone.

## Background

Invasive meningococcal disease (IMD), caused by the bacterium *Neisseria meningitidis,* is a serious and potentially life threatening disease, and a relatively rare but important cause of bacterial meningitis and sepsis [1-3]. IMD is endemic in Canada [3,4], with most cases caused by serogroups B (43.3%), C (25.1%), and Y (21.9%) in 2003 [4]. Since the last epidemic of serogroup A between 1940 and 1943, IMD caused by serogroup A has become extremely rare in Canada [5], and is associated with travel to areas where serogroup A is prevalent.

In Ontario, Canada’s most populous province (population 13.1 million), two meningococcal vaccines are currently in use at different ages. In September 2004, meningococcal C conjugate vaccine (MCCV) was introduced into a publicly-funded immunization program for children at one year of age (i.e. a toddler program). This was followed by a school-based program for grade 7 students (approximately 12 years of age). The toddler program is delivered by primary care physicians (family practitioners or paediatricians) and the school-based program is delivered by public health departments. In the fall of 2009, quadrivalent (serogroups A, C, Y, W135) conjugate meningococcal vaccine (MCV4) replaced MCCV for grade 7 students, while the toddler MCCV program remained unchanged.

The introduction of MCV4 into Ontario’s publicly-funded immunization program has left serogroup B as the only major IMD serogroup [3,6] still not vaccine preventable. Serogroup B IMD is of public health interest for a number of reasons. In 2004, an emerging clone of serogroup B *N. meningitidis* with sequence type (ST) 269 and an antigenic combination (serotype:serosubtype) of B:17:P1.19 was detected in Quebec [7,8], a province bordering Ontario; by 2005, 38 cases in Quebec were attributed to this strain [8]. In addition, vaccines targeting serogroup B have been difficult to develop due to their poor immunogenicity [9,10]. However, effective vaccines are currently in development and their anticipated approval has prompted an assessment of the epidemiology of serogroup B disease in Ontario.

Within this context, using reportable disease and laboratory data, we undertook a review of the epidemiology of serogroup B IMD in Ontario between 2000 and 2010 and estimated the number of infants needed to vaccinate (NNV) to prevent a case of disease.

## Methods

### Data sources

IMD is a legally reportable disease in Ontario. IMD case information including demographics (age, sex and health unit), serogroup, onset date, and available clinical data, is entered by 36 local public health units into the integrated Public Health Information System (iPHIS), the provincial reportable disease information system [11]. Once in iPHIS, cases are classified as confirmed if they meet the Ontario Ministry of Health and Long-term Care’s (MOHLTC) case definition [12]. During the surveillance period, the IMD case definition changed. Prior to April 28, 2009, clinical symptoms of meningitis with the identification of Gram negative diplococci from a normally sterile site (blood or cerebrospinal fluid [CSF]) or skin lesion, or with antigen detection from CSF or serum, and clinical symptoms of meningococcemia without laboratory confirmation, were included in the confirmed IMD case definition [13]. After this date, the case definition was more specific and required laboratory confirmation with the isolation of *N. meningitidis* or detection of its DNA by a validated nucleic acid amplification test from a normally sterile site [14].

IMD isolates from hospital and private laboratories in the province are sent to the Public Health Ontario Laboratories’ (PHOL) central laboratory, located in Toronto, Ontario, Canada, for confirmation of the species and serogrouping. Standard laboratory protocols are used for confirmation of *N. meningitidis*, and bacterial agglutination with rabbit anti-sera against capsular antigens is used for serogrouping. Once identified, isolates are sent to the National Microbiology Laboratory (NML), Public Health Agency of Canada (PHAC), Winnipeg, Manitoba, Canada, as part of national laboratory surveillance, and for strain typing. Information on *N. meningitidis* isolates obtained from PHOL includes patient demographics (age or year of birth, and sex), culture source (blood, cerebrospinal fluid, or other), serogroup, sender laboratory, and NML results (serotype, serosubtype, and multi-locus sequence type [MLST]).

### Record linkage

We linked confirmed IMD cases occurring between January 1, 2000 and December 31, 2010 that were reported in iPHIS to laboratory records via probabilistic record linkage. Linkage was done using four non-nominal common identifiers: age or date of birth (DOB), sex, serogroup, and diagnosing health unit. Onset and laboratory collection dates were used to verify matches. We coded sender laboratories in the PHOL records into health units based on laboratories’ postal codes within geographical jurisdictions of health units using the Public Health Unit Locator application (http://apps.publichealthontario.ca/PHULocator/). In most instances, age/DOB, sex, serogroup, and health unit were sufficient for record linkage. For laboratory records that could not be matched to iPHIS records, available epidemiologic and laboratory information was taken from laboratory requisitions.

### Descriptive analysis

We calculated incidence rates per 100,000 population using population denominators from Statistics Canada obtained through intelliHealth Ontario on July 6, 2011. Population denominators for 2010 were projected estimates. For geographical rates, as 2010 population denominators by health unit were not available at the time of analysis, we substituted 2009 population data to approximate 2010 population denominators. We analyzed data using EpiData version 2.2.1.171c, OpenEpi version 2.3.1, and SAS version 9.2 (SAS Institute, Cary, NC). Where appropriate, we excluded missing data from analyses and documented this; p-values ≤ 0.05 were considered significant.

### Number needed to vaccinate analysis

In anticipation of future availability of vaccine, we calculated a crude NNV to prevent a case of serogroup B meningococcal disease, using a modified number needed to treat formula [14]. We calculated the inverse of the infant (< 1 year) age-specific incidence multiplied by the expected vaccine efficacy using the observed annualized age-specific incidence among infants and a vaccine efficacy ranging from 70 and 80% and assuming only direct protection (no herd effects). This vaccine efficacy range was chosen to give a conservative estimate of the number needed to vaccine, as there are currently no published efficacy studies on novel meningococcal B vaccines. Because the vaccine will not protect very young infants, we also calculated the NNV assuming that only infant cases between 6 and 12 months were vaccine preventable (i.e., dividing the NNV by proportion of the infant cases that occurred among infants 6 to 12 months of age).

## Results

### IMD record linkage

Between 2000 and 2010, we identified a total of 713 unique IMD cases in either data source (both matched and unmatched). There were 682 confirmed IMD cases in iPHIS, and 582 unique meningococcal-related laboratory records in PHOL. Of the 682 confirmed cases in iPHIS, 551 (80.8%) were successfully matched to laboratory records in PHOL. Of all cases in iPHIS, 131 (19.2%) could not be matched to laboratory records; 90.8% (119/131) of these occurred prior to the change in case definition; of these 31.8% had serogroup information. Of all the cases in PHOL, 31 records (5.3%) could not be matched to cases in iPHIS.

### Descriptive epidemiology of serogroup B IMD cases

Over the 11-year period, a total of 259 IMD cases were caused by serogroup B, which accounted for the highest proportion of IMD cases (36.3%), followed by serogroup C (21.7%), serogroup Y (21.5%), and serogroup W135 (7.2%). Only three cases of IMD were caused by serogroup A. In addition to being the most common cause of IMD in Ontario, the proportion of IMD caused by serogroup B steadily increased, as the proportion of serogroup C decreased over the 11-year period, from 25.0% in 2000 and 2001 to 53.2% in 2008.

**Figure 1:**
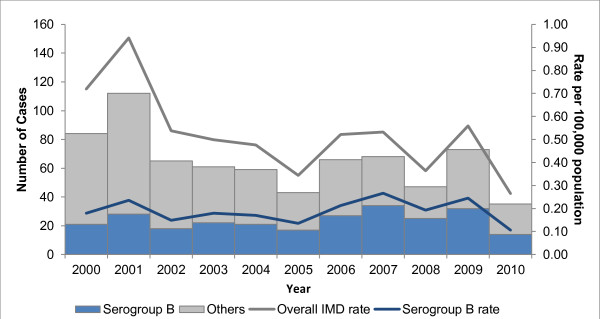
**Number and incidence (per 100,000) of IMD and by serogroup B, Ontario, Canada, 2000–2010.** The number of cases (solid bars) and annual incidence per 100,000 population per year (lines) for invasive meningococcal disease in Ontario, Canada, overall (all causes; N = 713) and by serogroup B (n = 259) in Ontario, Canada, from 2000 to 2010. IMD caused by other than serogroup B includes A, C, Y, W135, non-groupable and unknown.

Figure 1 shows the total number of IMD cases (overall and serogroup B) and incidence rates between 2000 and 2010. The annual incidence of serogroup B disease ranged between 0.11 per 100,000 population in 2010 and 0.27 per 100,000 population in 2007. This is in contrast to the annual rates of overall IMD which ranged between 0.26 in 2010 and 0.94 in 2001 per 100,000 population. While the incidence of overall IMD has decreased over time since its peak in 2001, there was no discernible change in the incidence of serogroup B disease over time.

**Figure 2:**
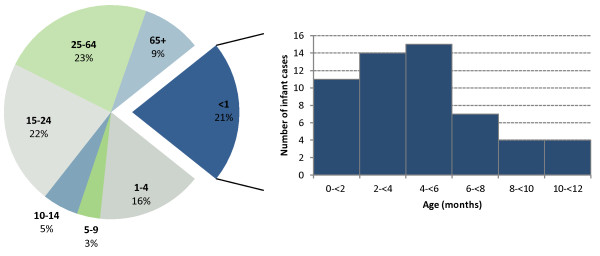
**Age distribution of serogroup B IMD cases, Ontario, Canada, 2000–2010 (N = 257).** Proportion (%) of serogroup B invasive meningococcal disease (IMD) cases by age group (N = 257) and number of infant serogroup B IMD cases by age (months) (N = 55) in Ontario, Canada, from 2000 to 2010. Two serogroup B IMD cases were excluded from analysis because of missing age information.

Of the serogroup B IMD cases with known sex information (256/259, 98.8%), 55.1% of the cases occurred in males with no significant variation over time (p = 0.32). The ages of serogroup B cases ranged between 13 days and 101 years, with a median age of 17 years. Although not a statistically significant change (p = 0.40), the median age increased from 5 years in 2000 to 20 years in 2010. The proportion of children (0–18 years) compared to adults (≥19 years) has decreased over the surveillance period from 70.0% in 2000 to 50.0% in 2010 (p = 0.03). Figure 2 shows the proportion of serogroup B cases in Ontario by age. Overall, 21% of cases occurred in infants less than 1 year of age (55 cases) and 16% occurred in young children aged 1 to 4 years (41 cases). By comparison, infants and children aged 1 to 4 years represented only 1.1% and 4.3% of Ontario’s population, respectively, in 2010. Most of the infant cases (72.7%) occurred in infants less than 6 months of age.

**Figure 3:**
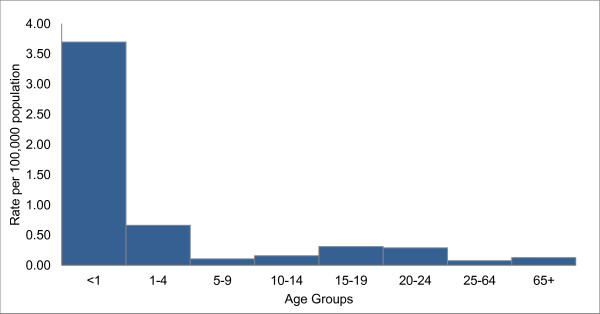
**Annualized age-specific incidence for serogroup B IMD**^**a**^**, Ontario, Canada, 2000–2010 (N = 257).** Annualized age-specific incidence per 100,000 population for serogroup B invasive meningococcal disease (IMD) in Ontario, Canada, for 2000 to 2010. ^a^Two serogroup B IMD cases were excluded from analysis because of missing age information.

As there was no statistically significant difference in median age over the 11-year period, and given the small number of cases, we determined annualized incidence rates by age between 2000 and 2010 (Figure 3). Infants less than 1 year of age had the highest rate at 3.70 per 100,000 population, followed by children aged 1 to 4 years.

There was some variation in rate of disease by health unit region. The region of Toronto had the lowest incidence (0.11 [95% CI 0.07 - 0.15] per 100,000 population) and was significantly lower than both the Central West (0.22 [95% CI, 0.17 - 0.28] per 100,000) and Eastern regions (0.22 [95% CI, 0.16 - 0.29] per 100,000). It is important to note that rates by region may be unstable due to small numbers.

### Case fatality

Table 1 shows the case fatality ratio (CFR) of serogroup B IMD overall and by age. The CFRs for serogroup C IMD is also shown for comparison. Of the serogroup B IMD cases with iPHIS records (242/259; 93.4%), the CFR during the period under surveillance was 10.7%. Because of the small number of deaths, CFR by age was grouped into the following: <1 year, 1 to 19 years, 20 to 64 years, and 65 years and older. Individuals aged 65 years and older had the highest CFR (21.1%), followed by infants (13.5%). Infants with serogroup B disease had a higher CFR than those with serogroup C disease, and the reverse was true among persons 1–19 years of age.

**Table 1:** Age-specific case-fatality ratio for IMD overall, and by serogroup (B and C), Ontario, Canada, 2000–2010

		**Age groups**				
		<1	1-19	20-64	65+	Overall
Serogroup B	# of deaths	7	4	11	4	26
	# of cases	52	85	86	19	242
	CFR (%)	13.5	4.7	12.8	21.1	10.7
Serogroup C	# of deaths	0	7	16	3	26
	# of cases	3	48	83	15	149
	CFR (%)	0	14.6	19.3	20.0	17.4

Age-specific case-fatality ratio (CFR) for serogroup B invasive meningococcal disease (IMD) cases in Ontario, Canada, from 2000 to 2010. The age-specific CFRs for serogroup C IMD are shown for comparison. Seventeen serogroup B IMD cases with only PHOL records were excluded from analysis because fatal outcome was unknown.

### Strain distribution of serogroup B IMD isolates

Table 2 shows the serotypes, serosubtypes and STs of serogroup B *N. meningitidis* isolates circulating in Ontario during the surveillance period. Of the serogroup B isolates with further strain characterization (212/259; 81.9%), there were a total of 56 unique antigenic variants (serotype:serosubtype). The most common antigenic variant was B:NT:P1.- (8.9%), following by B:14,19:P1.14 (6.6%), and B:NT:P1.14 (6.6%). Only three serogroup B cases in Ontario were antigenic variant B:17:P1.19/ST 269, the strain that was responsible for the emerging clone in Quebec. These cases occurred in 2003, 2006 and 2008.

**Table 2:** **Microbiological of *N. meningitidis *isolates in Ontario, Canada, 2000–2010 (N = 213) **

**Serotype**	**# of isolates (%)**	**Serosubtype**	**# of isolates (%)**	**ST**^**a**^	**# of isolates (%)**
4	39 (18.3)	P1.14	48 (22.5)	ST-5571	16 (11.3)
1	21 (9.9)	P1.4	29 (13.6)	ST-154	7 (5.0)
15	20 (9.4)	P1.9	25 (11.7)	ST-13	7 (5.0)
1,19	19 (8.9)	P1.16	12 (5.6)	ST-6617	6 (4.3)
14,19	16 (7.5)	P1.15	11 (5.2)	ST-6169	6 (4.3)
14	15 (7.0)	P1.7,16	9 (4.2)	ST-269	5 (3.5)
19	7 (3.3)	P1.13	8 (3.8)	ST-275	4 (2.8)
17	3 (1.4)	P1.2,5	5 (2.3)	ST-136	4 (2.8)
15,19	1 (0.5)	P1.6	4 (1.9)	ST-60	4 (2.8)
		P1.19	4 (1.9)	ST-336	4 (2.8)
		P1.5	3 (1.4)	ST-32	4 (2.8)
		P1.7	3 (1.4)	ST-41	4 (2.8)
		P1.12	3 (1.4)	ST-1578	3 (2.1)
		P1.10	3 (1.4)	ST-43	3 (2.1)
		P1.2	2 (0.9)	ST-1946	3 (2.1)
		P1.1,7	2 (0.9)	ST-6614	3 (2.1)
		P1.13,19	1 (0.5)	ST-461	2 (1.4)
		P1.14,19	1 (0.5)	ST-568	2 (1.4)
		P1.15,19	1 (0.5)	ST-571	2 (1.4)
				ST-6686	2 (1.4)
				ST-35	2 (1.4)
				ST-570	2 (1.4)
				Others^d^	46 (32.6)
NST^b^	72 (33.8)	NSST^c^	39 (18.3)		
Total	213 (100.0)	Total	213 (100.0)	Total	141 (100.0)

Serotypes, serosubtypes, and sequence types of serogroup B *N. meningitidis* isolates circulating in Ontario between 2000 and 2010. Twenty-nine isolates were excluded because further strain characterization was not done. Table abbreviations: ^a^ST, sequence type; ^b^NST, nonserotypeable; ^c^NSST, nonsubserosubtypeable; ^d^Others includes another 46 unique STs, each representing 0.7% of isolates with MLST information.

#### Number needed to vaccinate

Using the infant annualized age-specific rate of 3.7 per 100,000 population and a vaccine efficacy ranging from 70 and 80%, we would need to vaccinate between 33,784 and 38,610 infants to prevent one case of disease, assuming that all cases under one year would be preventable by vaccination. However if we assume that infant cases under 6 months of age (72.7% of cases) may not be vaccine preventable, the NNV will increase to between 123,751 and 141,429 infants.

## Discussion

As IMD is a relatively rare disease and reporting is mandatory in Ontario, it was possible to link reportable cases to laboratory records via probabilistic linkage. The probabilistic linkage of case-level records in iPHIS and PHOL had a 77.3% (551/713) success rate and provided additional case information that enhanced our ability to describe the epidemiology of serogroup B disease in the province. While the record linkage of iPHIS to PHOL records remains incomplete (19.2% iPHIS records and 5.3% unique PHOL records were unmatched), it is not unexpected to have mismatches for a number of reasons. First, both data sources are passive systems and thus rely solely on healthcare providers, public health providers, and laboratories reporting IMD cases and forwarding *N. meningitidis* isolates to the public health laboratory for serogrouping. Second, prior to the change in case definition on April 1, 2009, confirmed IMD cases were based on the presentation of relevant clinical symptoms of meningococcemia both with and without laboratory confirmation [13]. Third, non-viable isolates from hospital laboratories could have been genogrouped using polymerase chain reaction and these would not be received at PHOL. Fourth, we did not have access to nominal data for this analysis and this limited our abilities to improve record linkage through deterministic linkage.

While it is difficult to determine whether our data is a true representation of all serogroup B cases in Ontario, we expect that most cases are captured either through the reportable disease information system or PHOL data given that this disease is associated with high public dread, has extremely high probability of hospitalization and microbiologic testing of the infected individual, and because PHOL is the only laboratory in the province that provides serogrouping of viable isolates.

In the last 11 years, as the incidence of serogroup C has decreased serogroup B has accounted for a greater proportion of IMD cases and has become the most prevalent cause of IMD in Ontario. The increase in the proportion of serogroup B IMD coincides with the introduction of MCCV into the provincially funded routine childhood immunization program in 2004 and a subsequent decrease in the incidence of serogroup C disease among all age groups; this should not be interpreted as an increasing rate in serogroup B disease. Our data were consistent with the natural fluctuation of the disease [4] and also suggests that there were no predominant strains emerging in the province. As there was a change in case definition during the period under surveillance (i.e., April 2009), and the revised case definition was more specific, this could have resulted in a small decrease in reported cases in 2009 and 2010.

Consistent with the national data [4], our findings reveal that people with serogroup B disease tend to be young, with infants having the highest rates. Infants represented only 1.1% of Ontario’s population in 2010 yet accounted for 21% of the overall disease burden with the majority (72.7%) of cases in younger infants less than 6 months of age. This will have important implications for vaccination programs once a vaccine that protects against this serogroup becomes available. Decision-makers will need to consider the age of vaccination and the number of cases that will be preventable through vaccination, as well as possible herd immunity effects. With an infant vaccination program, the age group with the highest incidence could be targeted, but cases occurring in very young infants will not be preventable unless they are protected by herd immunity, as has been demonstrated for serogroup C immunization. Given that novel meningococcal B vaccines are based on sub-capsular proteins rather than polysaccharides, and there are no published efficacy or effectiveness studies on these vaccines, we do not yet know whether these vaccines would result in herd immunity as observed from MCCV and other bacterial conjugate vaccines. The increase in median age throughout the surveillance period was of interest despite the lack of statistical significance, and is worth monitoring in the future, as changes in age distributions of serogroup B IMD can further impact vaccine decision-making.

Our crude NNV to prevent a single case of disease is high, in excessive of 30,000 infants, yet this is conservative as it assumes that all cases under one year would be vaccine preventable. For the calculation we used a vaccine effectiveness of between 70 and 80%, yet this is an assumption as the true value is not known. As noted in our results, approximately 70% of our infant cases occurred among infants under 6 months of age and these cases may not be vaccine preventable depending on age at vaccination and duration of time to mount an immune response. In a phase IIb clinical trial, Gossger and co-authors [15], found that a schedule of three doses of Novartis’ novel multicomponent meningococcal B vaccine (4CMenB) given to infants at 2, 4, and 6 months of age, and in an accelerated schedule at 2, 3, and 4 months of age were necessary to achieve optimal immunogenicity. This would suggest that disease in infants less than 6 months of age, using a 2, 4, and 6 month schedule, which is typical in Canada, may not be vaccine preventable. Using this assumption the revised NNV would increase to over 120,000 infants. Although applying the number needed to treat concept to vaccines is not new, there is no agreed upon NNV threshold for vaccine decision-makers.

### Limitations

Given the rarity of the disease, the rates or proportions that we calculated may be unstable due to small numbers and therefore the results should be interpreted with caution. Our dataset was substantially limited in the amount of clinical data collected despite these fields being included in the reportable disease system, highlighting the importance of ensuring the collection of all relevant clinical data for reportable diseases including type of syndrome and presence of complications, to allow for a better understanding of the burden. As there are currently no licensed meningococcal B vaccines in Canada, our crude NNV calculations were based on several assumptions including vaccine efficacy, vaccine uptake and no herd immunity and is meant to give a conservative estimate until these parameters are better defined, if and when a vaccine becomes available.

## Conclusions

Although rare, serogroup B meningococcal disease is endemic in Ontario and is greatly feared by the public. This analysis will provide important information for future decision-making regarding the inclusion of serogroup B vaccines in the publicly funded immunization program. Although the vaccines are highly anticipated, our number needed to vaccinate analysis suggests that these decisions will be difficult.

## Abbreviations

IMD: Invasive meningococcal disease; NNV: Number needed to vaccinate; CFR: Case-fatality ratio; MCCV: Meningococcal C conjugate vaccine; MCV4: Quadrivalent meningococcal vaccine (serogroup A, C, Y and W135); ST: Sequence type; iPHIS: Integrated Public Health Information System; MOHLTC: Ontario Ministry of Health and Long-term Care; CSF: Cerebrospinal fluid; DNA: Deoxyribonucleic acid; PHOL: Public Health Ontario Laboratory; NML: National Microbiology Laboratory; PHAC: Public Health Agency of Canada; MLST: Multi-locus sequence type; DOB: Date of birth; 4CMenB: Four component meningococcal B vaccine (Novartis).

## Competing interests

The authors declare that they have no competing interests and have received financial support from Public Health Ontario only for this study.

## Authors’ contributions

SLD and SW contributed to the overall design of the study. RSWT provided the serotyping, serosubtyping and MLST of the isolates. SW extracted laboratory data from PHOL. FBJ and PR supported queries regarding laboratory data from PHOL. KJ extracted and supported queries regarding data from iPHIS. VD was responsible for the record linkage of the two data sources and the compilation, validation, and analysis of the data. SLD, FBJ and SW supported the record linkage, and analysis of the data. SLD, NSC, FBJ contributed to the validation and interpretation of the results. VD and SLD wrote the manuscript. All authors contributed to the editing of the manuscript and have reviewed and approved the final version.

## Author information

VD completed this study at Public Health Ontario as part of the Dalla Lana School of Public Health, University of Toronto, practicum requirements for the Master of Public Health in Epidemiology degree.

## Pre-publication history

The pre-publication history for this paper can be accessed here:

http://www.biomedcentral.com/1471-2334/12/202/prepub
